# When information security depends on font size: how the saliency of warnings affects protection behavior

**DOI:** 10.1080/13669877.2022.2142952

**Published:** 2022-11-14

**Authors:** Nico Ebert, Kurt A. Ackermann, Angela Bearth

**Affiliations:** a Center for Process Management & Information Security, Institute of Business Information Technology (IWI), ZHAW SML, Winterthur, Switzerland; b Center for Behavioral Insights & Pricing, Institute of Marketing Management (IMM), ZHAW SML; c Consumer Behavior, Institute for Environmental Decisions (IED), ETH Zurich

**Keywords:** Information security, saliency, warnings, fear appeal, digital risk, cookies, cookie banners

## Abstract

Prior research on how to improve the effectiveness of information security warnings has predominantly focused on either the informational content of warnings or their visual saliency. In an online experiment (*N* = 1’486), we disentangle the effect of both manipulations and demonstrate that both factors simultaneously influence decision making. Our data indicate that the proportion of people who engage in protection behavior can be increased by roughly 65% by making a particular warning message more visually salient (i.e. a more conspicuous visual design is used). We also show that varying the message’s saliency can make people behave very differently when confronted with the same threat or behave very similarly when confronted with threats that differ widely in terms of severity of outcomes. Our results suggest that the visual design of a warning may warrant at least as much attention as the informational content that the warning message conveys.

## 1. Introduction

Warning individuals about potential adverse consequences of their online behavior via persuasive messages is a common practice in information security (ISec). Security warnings are intended to help protect users from threats. For instance, web browser warnings are supposed to help protect users from malware, phishing, and network attacks (Reeder et al. 2018). The use of warning messages has also been discussed in related areas, such as warning consumers about biases in product recommendation agents (Xiao and Benbasat 2015). Despite their widespread use, research has consistently shown that warnings are often ineffective, not just in information security (Dhamija, Tygar, and Hearst 2006; Sotirakopoulos, Hawkey, and Beznosov 2011) but also for health or environmental hazards (Bearth et al. 2020; Boelhouwer and Davis 2010). Habituation, which is a ‘decreased response to repeated stimulation’ (Groves and Thompson 1970, p. 419), has been identified as a key factor that helps to explain why adherence to warnings drops over time (Anderson, Vance, et al. 2016). Even warnings that the user has not seen before may be subject to habituation based on prior experiences with similarly looking notifications (Anderson et al. 2017; Vance et al. 2019). Moreover, a lack of risk awareness (e.g. linked to the lack of knowledge about digital risks or low initial risk perception elicited by the product or service) might lead users to disregard warnings, despite being exposed to them (Visschers et al. 2012; Wogalter and Mayhorn 2005).

It has been demonstrated that continuously changing the visual appearance of warnings can help reduce the effect of habituation and consequently also mitigate the decline in adherence (Anderson, Jenkins, et al. 2016; Anderson, Vance, et al. 2016; Kirwan et al. 2020; Vance et al. 2018). Apart from visual appearance, other factors have also been shown to affect how people react to warnings. For example, researchers have used fear appeals, a specific form of warning intended to evoke fear, to demonstrate that different levels of threat (e.g. low vs. high risk) described in the messages can lead to different levels of individual protection behavior (Boss et al. 2015). These findings suggest that not only the visual appearance of an information security warning message but also its factual content affect individual protection behavior.

So far, the available research does not provide a theoretic account for how these factors *simultaneously* influence decision making – neither with respect to their relative effect sizes nor regarding the path of influence, i.e. whether the effects act sequentially or in parallel. This is an important issue as one factor cannot be designed without the other. For instance, research in visual and decision neuroscience suggests that visual information can evoke decision-making biases (Conzola and Wogalter 2001; Krajbich, Armel, and Rangel 2010; Krajbich and Rangel 2011), which can lead users to make choices based on the choice alternatives’ visual attributes rather than their consequences. This raises the question if the decision to protect oneself based on an information security warning message may even be affected more by visual attributes, such as font size or color, than by relevant textually described threats, as research in other domains may suggest (e.g. Buchmüller et al. 2022; Visschers et al. 2012).

This study investigates the role of two basic factors of security warnings – namely the informational content (i.e. the factual information describing the actual threat that the warning refers to) and its visual saliency (i.e. the visual conspicuousness of the text conveying the informational content) – in decision making. These basic factors are not unique to information security but relate to other warnings as well, such as warnings on household chemicals (type of hazard and pictogram style) or health warnings on cigarette packages (the content of health warning and picture used on the packaging). We rely on the concept of fear appeals, which is already established in the fields of psychology, risk research, and ISec (Boss et al. 2015; Tannenbaum et al. 2015; Visschers et al. 2012), as well as on the concept of saliency, which is a key concept in human visual cognition (Itti 2007), to investigate whether both the informational content and its visual saliency *simultaneously* affect decision making. To gain empirical evidence, we conduct an online ‘lab-in-the field’ experiment. This allows us to recruit a broad range of subjects that use their own devices to increase external validity (Karahanna et al. 2018). To facilitate this research, we examine a specific type of threat, namely cookies, which are a widely used form of online tracking on many websites. Cookie banners ask users to accept tracking practices. However, relevant information concerning tracking practices is often of low visibility in these cookie banners (Bornschein, Schmidt, and Maier 2020). To study the role of warning content and its visual saliency, we enriched cookie banners with fear appeals. The primary research question we address in this study is as follows: *To what extent do the informational content and the visual saliency of a textual warning affect the decision to accept or decline the use of cookies?*

## 2. Background and related work

### 2.1. Study context

One of the most virulent threats that people are regularly exposed to when using apps and visiting websites is the threat of privacy intrusions (Englehardt and Narayanan 2016). However, most people do not protect themselves sufficiently against this kind of threat for a variety of reasons, one of which may be that they are not sufficiently made aware of it by service providers in the first place (Degeling et al. 2019). In the past, privacy regulators have typically asked organizations for general ‘transparency’ regarding data processing towards individuals without specific requirements to emphasize risks or explicitly warn individuals regarding privacy risks. Modern data protection laws around the world are built on ‘fair information practices principles’ (FIPPS) (Cate 2006). The FIPPS, which were first developed in the 1970s, demand that organizations establish transparency in their data processing practices (Gellman 2017). In later versions, informing the consumer (i.e. the notice principle) was treated as the most fundamental principle of FIPPS, and essential information requirements were defined accordingly (e.g. personal data collected and processing purposes). The European Union’s General Data Protection Regulation (GDPR) goes even further by explicitly prescribing categories of privacy information that must be disclosed to consumers (Gellman 2017). As another example, since 2020 the California Consumer Privacy Act of 2018 has required businesses to place a ‘clear and conspicuous’ link on their business websites titled ‘Do Not Sell My Personal Information’ (California Consumer Privacy Act of 2018 2018, 2020). This is intended to enable consumers to opt-out of the implicit agreement that a company is allowed to sell their personal information.

Researchers have studied the effectiveness of various approaches intended to lead to greater individual privacy awareness. Privacy policies constitute the traditional approach to mitigating individual uncertainty and have been studied extensively (Schaub, Balebako, and Cranor 2017). However, the privacy policy approach has many downsides, with one being that privacy policies are often ‘click-wrapped’ behind links that are seldom clicked (Ebert, Ackermann, and Scheppler 2021; Obar and Oeldorf-Hirsch 2020). Alternative ways to display privacy policy information have been developed that promise better user awareness of information relevant for privacy-related decisions. For instance, privacy ratings for e­commerce websites have been shown to be able to influence behavior toward higher levels of self-protection (Tsai et al. 2011). As another example, very short privacy statements have been proposed as an alternative to displaying complete privacy policies. Information preferences of users regarding such brief statements have been collected and have indeed been shown to create higher user awareness (Ebert, Ackermann, and Heinrich 2020, Ebert, Ackermann, and Scheppler 2021).

In our research design, we go beyond the usual principle of informing users of privacy practices (which might not include risks) and try to actively warn them to avoid threats. Specifically, we use short persuasive messages pointing to threats caused by browser cookies on websites to influence the choice to accept or decline cookies. Cookies can be used to track user behavior, and in 2009, the ‘EU Cookie Directive’ (2009/136/EC) changed Article 5(3) of the ePrivacy Directive (2002/58/EC) to state that ‘the storing of information … in the terminal equipment of a … user’ is only allowed if the individual ‘has given his or her consent, having been provided with … information … about the purposes of the processing’ (DIRECTIVE 2009/136/EC OF THE EUROPEAN PARLIAMENT AND OF THE COUNCIL 2009). This led to websites worldwide moving to prominently display consent notices (referred to as ‘cookie banners’), informing users about the use of cookies and asking for their consent. Despite its name, the EU Cookie Directive covers all forms of online tracking technology (such as device fingerprinting, for instance) and thus does not only apply to cookies in the narrow sense. However, while many websites are forced to display cookie banners, the way in which these cookie banners are visually designed is hardly regulated at all. At the same time, website owners clearly have an incentive to increase the number of visitors who accept cookies and therefore also use means of visual design (Cofone 2017).

An empirical analysis of popular websites in 28 EU countries showed that 62% implemented cookie banners after the European Data Protection Regulation came into force in May 2018. It turned out that many of these websites used malicious user interface designs (‘dark patterns’) in their cookie banners to evoke user consent (Degeling et al. 2019). A series of field experiments with 80,000 users in Germany demonstrated that small design changes in cookie banners can have large effects on the decision to accept or decline the use of cookies (Utz et al. 2019). In another field experiment in Germany with close to 1,500 users, the consent rate increased when the accept button was designed differently than the decline button and when the benefits of cookies were framed more positively (Bauer, Bergstrøm, and Foss-Madsen 2021).

In contrast to the industry practice of using visual design to persuade users to accept cookies, in our study we use visual design changes not to hide the risks associated with cookies, but to highlight them based on the concepts of fear appeals and visual saliency.

### 2.2. Theoretical foundations

#### 2.2.1. Fear appeals

A fear appeal is a specific form of warning message intended to evoke fear. Fear appeals typically not only describe a threat but also provide a recommended response to the threat (Rogers 1983; Rogers and Deckner 1975). The Protection Motivation Theory (PMT) is the primary theoretical foundation for studies investigating the potential of fear appeals to alter security behavior (Wall and Buche 2017) and has been also applied in privacy-related studies (e.g. Albayram et al. 2017; Meier et al. 2020; Mousavi et al. 2020).

Within PMT, a fear appeal is a stimulus designed to trigger fear as well as threat and coping appraisal processes, which leads to a protection motivation and ultimately to a behavioral change (Floyd, Prentice‐Dunn, and Rogers 2000). Fear is ‘aroused in response to a situation that is judged as dangerous and toward which protective action is taken’ (Rogers 1975). The underlying theoretical assumption is that the fear appeal first triggers threat-appraisal processes, in which *perceived threat severity* (1), *perceived threat vulnerability* (2), and generated *fear* (3) inspire protection motivation and must outweigh *maladaptive rewards* (4) not to engage in protection motivation. Subsequently, a coping appraisal process is started in which individual *response efficacy* (5) and *self-efficacy* (6) must outweigh *response costs* (7) for engaging in the *protection motivation* (8). Protection motivation then leads to a specific behavior (9): either an adaptive response (i.e. a behavioral change to control danger) or maladaptive mode (i.e. no behavioral change). The PMT with the abovementioned constructs (1)–(9) based on research by Floyd, Prentice‐Dunn, and Rogers (2000) and Rogers and Prentice-Dunn (1997) was introduced to ISec research by (Boss et al. 2015) as the ‘full nomology’ of PMT.

On the one hand, researchers have pointed out that threatening communication in fear appeals can have no effect or may even backfire in situations with low efficacy, for example when individuals have no sufficient strategies to cope with threats (Boss et al. 2015; Peters et al. 2018; Ruiter et al. 2014). On the other hand, it has recently been proposed that evoking fear as a negative emotional arousal via threatening communication is to some extent even necessary to trigger coping behavior in the first place (Zhang and Borden 2020). Moreover, ISec research has sometimes reported contradictory findings over the years regarding which constructs actually drive protection motivation and so the field constitutes an active area of research (Schuetz et al. 2020; Wall and Buche 2017). For example, some studies have reported that protection motivation is driven by threat severity (Boss et al. 2015), while others have not (Menard, Bott, and Crossler 2017). Moreover, some research suggests that highly emotional information may either increase or decrease risk appraisal depending on prior beliefs (Thalmann and Wiedemann 2006). Differences in audiences (personal vs. organizational users) and fear appeal content (abstract vs. concrete messages, personally relevant messages) have been identified as influential factors and therefore potential explanatory accounts for differences among studies (Johnston, Warkentin, and Siponen 2015, Johnston et al. 2019; Schuetz et al. 2020). Also, gain and loss framings of security messages (Seo and Park 2019) or the choice of particular warning signal words (Hellier et al. 2007) have been shown to potentially affect users’ intentions to protect themselves from corresponding risks.

Findings like these demonstrate that informational content is an important factor affecting protection behavior. However, research has shown that the effects of risk communication do not only depend on the content of the communication but also on its design features and the format in which it is presented. Some research even suggests that so-called incidental affect induction (i.e. through the format and context in which a message is presented) may be more influential than integral affect induction (i.e. through the message itself) in risk communication (see e.g. Visschers et al. (2012)). As we discuss next, visual saliency may be a particularly important general design feature in this respect and play a crucial role regarding risk appraisal and protection behavior in response to a risk-communicating warning message.

#### 2.2.2. Visual saliency

Visual saliency is a concept originating from cognitive and perceptual psychology as well as visual neuroscience; it refers to the degree to which an item stands out in contrast to other items in its vicinity (Itti 2007). For example, among an arrangement of 100 arrows pointing to the left, one arrow pointing to the right would stand out and could thus be termed salient. This concept is based on the assumption that the human visual apparatus features ‘saliency maps’ representing the distinctiveness of objects in the visual field (Itti and Koch 2001; Li 2002; Treisman and Gelade 1980). In line with these theoretical assumptions, highly salient items are more likely to attract attention (Itti and Koch 2001; Theeuwes et al. 1998) and are consequently also more likely to be perceived consciously than less salient items (Hoffman and Singh 1997; Reynolds and Desimone 2003; Theeuwes et al. 1998). The underlying cause for this visual saliency effect appears to be that more salient visual stimuli evoke longer eye fixations than less salient stimuli (Henderson, Weeks, and Hollingworth 1999; Itti and Koch 2001; Mannan, Kennard, and Husain 2009; Parkhurst, Law, and Niebur 2002).

Due to its potential to direct attention, visual saliency is a highly relevant concept in the context of human-computer interaction research, information display optimization, and interface design in general (Jarvenpaa 1990; J. D. Still, Hicks, and Cain 2020; J. D. Still and Masciocchi 2010; J. Still and Still 2019). For instance, Veas et al. (2011) showed that modulating the saliency of visual regions in a video can shift the video spectators’ attention and influence corresponding recall performance. Another study demonstrated that privacy information in apps was better recalled when made salient and presented exclusively rather than alongside related context information (Ebert, Ackermann, and Scheppler 2021).

The visual saliency effect affects more than just attention and recall, however. Moreover, it appears to affect choice behavior because stimuli that evoke more attention also appear to be perceived as more valuable by the cognitive system (Armel, Beaumel, and Rangel 2008; Armel and Rangel 2008; Krajbich, Armel, and Rangel 2010; Shimojo et al. 2003). Consequently, under certain conditions, visual saliency can alter preferences at the moment of choice and lead people to select the more salient out of two options, while they would have preferred the other option under conditions in which neither option is more salient than the other (Krajbich, Armel, and Rangel 2010; Krajbich and Rangel 2011; Milosavljevic et al. 2012; Rangel, Camerer, and Montague 2008; Shimojo et al. 2003). Consequently, decisions may be modulated by attending to saliency as a design feature that can be used in choice architecture, i.e. construing and designing a choice context in a way that predictably promotes the choice of a particular alternative (Thaler and Sunstein 2009).

In comparison to the previously mentioned research on saliency effects, we do not manipulate the saliency of options from which people can choose. Rather, we manipulate the visual saliency of information that may be relevant for making a corresponding choice in the first place. Concretely, we manipulate the visual saliency of fear appeal messages on cookie banners in which the messages are displayed and assess the effect this may have on cookie decline rates. In doing so, we extend previous research on visual saliency effects and evaluate the effect of a fear appeal message’s visual saliency in comparison to the fear appeal message’s informational content. Concretely, we manipulate visual saliency by simply altering the font size of the warning text conveying the threat and manipulating informational content by referring to a severe or harmless threat, respectively.

## 3. Hypotheses

Drawing from protection motivation theory, we posit that a security warning describing a high level of threat compared to a low level of threat leads to higher levels of perceived threat and fear, which in turn lead to an increased protection behavior – given that response-efficacy and self-efficacy outweigh response costs (Floyd, Prentice‐Dunn, and Rogers 2000; Rogers and Prentice-Dunn 1997). Therefore, we state the following first hypothesis:

*Hypothesis 1: Participants exposed to fear appeals that describe a high threat are more likely to protect themselves compared to participants exposed to fear appeals that describe a low threat.*

Research on visual saliency suggests that visual objects with highly salient features are more likely to attract attention (Itti and Koch 2001; Theeuwes et al. 1998) and can ultimately modulate behavior (Thaler and Sunstein 2009). There are several features an object can have that may make it stand out from its environment and thus increase its saliency. For instance, the saliency of an object’s particular part or component may depend on its color or orientation (Wolfe and Horowitz 2017). An example is a red object among other grey objects. Importantly, however, there are also certain features of an object that are unlikely to guide visual attention, such as letters (Wolfe and Horowitz 2017). In the context of our study, this means that text per se (e.g. a warning message) within other, not warning-related text, is unlikely to affect visual attention.

One feature of an object that can clearly make it stand out is the object’s relative size (Wolfe 2014; Wolfe and Horowitz 2017). Therefore, in line with existing research on warnings (Braun, Silver, and Stock 1992; M. Wogalter and Mayhorn 2005), we expect that a security warning text in large font size will be more salient and thus attract more attention than a warning in small font size. A more salient warning text is therefore more likely to be consciously noticed by participants and consequently more likely to influence their decisions to protect themselves (Hoffman and Singh 1997; Reynolds and Desimone 2003; Yantis 2005). As a result, we expect that a more salient warning will be considered a more relevant stimulus by participants (Armel, Beaumel, and Rangel 2008, p. 208; Armel and Rangel 2008; Krajbich, Armel, and Rangel 2010; Shimojo et al. 2003) and lead to higher protection rates. Hence, based on the described research on visual saliency, we state the following second hypothesis

*Hypothesis 2: Participants exposed to fear appeals with highly visually salient content are more likely to protect themselves than participants exposed to fear appeals with less visually salient content.*

Cognitive psychology suggests that perception is a prerequisite for the recognition of objects (e.g. understanding the meaning of the text) (Goldstein 2010, p. 8). Therefore, we can assume that the saliency of a warning content is a prerequisite for its interpretation, including the evaluation of the corresponding level of threat. Saliency can therefore be expected to moderate the effect of threat on protection behavior. We, therefore, state the third hypothesis:

*Hypothesis 3: The visual saliency of a warning content moderates the effectiveness of the corresponding threat regarding threat appraisal and finally protection behavior: A threat should have a bigger effect on threat appraisal and consequently protection behavior, the more salient the content is in which the threat is conveyed.*

## 4. Methodology

### 4.1. Experimental design

The empirical part of our study consisted of three elements: (1) a pre-study preceding the experiment to determine which cookie tracking practices might pose a threat and could therefore be included in the subsequent experiment; (2) the online ‘lab-in-the-field experiment’ to investigate how the saliency of fear appeals affects privacy behavior, and (3) a subsequent survey following the experiment to conduct the manipulation checks and to collect demographic information. Our Institutional Review Board evaluated the study as ethically sound, and all participants gave informed consent before participation. After the experiment, participants were truthfully informed about the study’s purpose and received a small financial compensation. We did not collect personal information (e.g. IP addresses).

#### 4.1.1. Pre-study: Cookie tracking survey

We developed an initial pre-study, carried out in the UK, the purpose of which was to elicit major concerns that individuals have with regard to different types of cookie tracking practices used by websites (cf. Appendix A). This allowed us to design an experiment to test the effectiveness of a fear appeal that addresses a relevant concern.

One of the cookie tracking practices that turned out to raise a particularly high level of concern among users was ‘session replay’. Session replay tools are widely used tools allowing the owner of a website to record a video of the mouse movements, clicks, and keypresses of any user who is visiting that website (Englehardt, Acar, and Narayanan 2017). While these tools are used to optimize a website’s usability, they can capture sensitive user inputs even before a user consciously submits data to the website (e.g. a password, credit card number, or search term incidentally entered in an online form). As session replay tools are typically provided by third parties as a service and integrated into existing websites, sensitive data might be shared with these third parties, too.

#### 4.1.2. Online experiment: Design of website and fear appeals

To measure the effect that the visual saliency of a fear appeal has on privacy behavior, we specifically designed a context for the online experiment in which protection motivation can arise. Based on the full nomology of PMT, this is the case if a) a threat is detected, b) maladaptive rewards are not greater than the threat, and c) efficacy must be greater than response costs for an adaptive response (Floyd, Prentice‐Dunn, and Rogers 2000; Rogers and Prentice-Dunn 1997).

In practice, information on threats is typically absent from cookie banners because websites have an incentive to evoke users’ consent to be tracked, while the regulators do not provide users with warnings of threats associated with cookie tracking (Cofone 2017). Maladaptive rewards can include the benefits of a personalized website and personalized advertisements on a website. In practice, website owners refer to these benefits with statements, such as ‘We use cookies to improve the website experience’, to convince users to accept cookies (Bauer, Bergstrøm, and Foss-Madsen 2021). Response costs of declining the use of cookies are often higher than the costs of accepting cookies. Previous studies have found that many websites make use of malicious user interface patterns to increase response costs (e.g. number of clicks, required time, cognitive costs) (Utz et al. 2019).

In our experiment, a cookie banner containing a fear appeal was implemented in a fictitious adult shopping website we dubbed ‘amorini.co.uk’, which pretended to sell sex toys. The context of an adult website was chosen due to the assumption that privacy may be a particularly important factor for users interacting with such a website.

When participants entered the website, the cookie banner was shown immediately as a pop-up window while the rest of the page was disabled and grayed out. The banner informed participants that the website they were about to enter would use cookies. To reduce maladaptive rewards, the banner did not mention specific benefits associated with accepting cookies. Participants then had to accept or decline the use of cookies by clicking the appropriate button to reach the website, as there was no other option to close the user dialog. To lower response costs for declining the use of cookies compared to real-life settings, both buttons were equally salient and both declining and accepting cookies required the same number of clicks, namely only one. Also, both buttons were designed in the same subtle gray color. Once the users had clicked one of the two buttons, the adult website was presented and suggested that the user allow personalized product recommendations. The design of the website (cf. Appendix B) used subtle visual language that contained no explicit content and gave the impression of a real, professional provider.

The fear appeal on the cookie banner was implemented in this study as if it was issued by a third party (e.g. a browser extension). The text component was constructed in a way that described a threat and at the same time also suggested a corresponding coping opportunity (‘…press “Decline”’, Table 1). The signal word ‘warning’ was used in combination with the pictorial symbol of an exclamation mark in parentheses (‘(!)’) to attract attention. Furthermore, red was selected as the text color of the fear appeal and the fear appeal was placed at the top of the cookie banner to make it highly visible (Wogalter, Conzola, and Smith-Jackson 2002).

The study involved a 2 × 2 experimental design, resulting in four treatment conditions (Table 1). As the first independent variable *saliency*, we manipulated the degree to which the fear appeal was visually salient with two levels (low vs. high). We operationalized visual saliency in terms of font size, and participants in the low saliency condition were presented with the text in 85% scaled font size relative to the regular cookie banner text, while participants in the high saliency condition were presented with the text scaled to 160%. As the second independent variable *threat*, we manipulated the informational content of the warning message in terms of the severity of the threat that was referred to by the fear appeal, again with two levels (low vs. high). In the low-threat condition, participants were presented with a fear appeal referring to a harmless threat, thereby conveying irrelevant information intended to raise as little fear as possible, while the participants in the high-threat condition were warned about the practice of session replay with third-party data sharing, which was identified in the pre-study as a data practice that raises particularly high concerns.

### 4.2. Sample

Recruitment took place via the online panel provider Prolific and addressed UK-based residents as participants for this study. We recruited a random sample and did not prescreen participants for privacy sensitivity to rule out a systematic bias in the estimation of cookie decline rates. As a requirement for participation, participants had to use their own devices. This was important because we wanted to increase external validity by increasing the likelihood that the fear appeal on the website presented would be perceived as a legitimate and personal threat. Due to specific required screen dimensions, only participants using desktop computers and laptops were accepted for participation in the study; participants with tablets and smartphones were excluded. Of 1599 participants that initially completed the study, 83 were excluded because they failed an attention check and 30 were excluded because they completed the entire instrument in less than 50% of the median completion time of 6 minutes (Greszki, Meyer, and Schoen 2015). This resulted in a final sample of *N* = 1486 participants.

### 4.3. Experimental task and procedures

The experiment was carried out via an online survey software in which we embedded the stimulus website. The steps in the experiment were as follows: (1) participants were asked for a ‘usability comparison between two websites’ and randomly assigned to one of the five conditions (no fear appeal [control], low saliency/low threat, low saliency/high threat, high saliency/low threat, high saliency/high threat); (2) To familiarize participants with the experimental setting and to increase the realism they were first redirected to an existing, popular UK housing website and asked to search for a flat; (3) After thirty seconds, participants could move on and were then asked to search for products on the fictitious website www.amorini.co.uk; (4); thirty seconds later they could move on to answer several survey questions regarding their perception of the presented stimulus material (manipulation checks), and (5) to provide demographic information.

### 4.4. Measurement of the dependent variable

The focal dependent variable, that is, the cookie decline rate, was simply measured by the number of participants who declined the use of cookies as opposed to accepting cookies or deciding not to interact with the cookie banner.

### 4.5. Pre-test

A pre-test was conducted with eight subjects before the main experiment to ensure that the stimulus material and survey items were comprehensible. Additional technical tests were conducted to ensure that the stimulus website was working adequately with typical web browsers and screen resolutions.

## 5. Results

### 5.1. Demographics

The gender distribution was slightly asymmetric, with females constituting 58% of the participants in our study. The age of the participants ranged from 18 to 84 (M = 36.6, SD = 13). Importantly, there were no statistically significant differences concerning the distributions of demographic variables across the five experimental groups. Appendix C informs about the participants’ characteristics per condition in more detail.

### 5.2. Manipulation checks

Table 2 outlines the manipulation checks that were performed after the stimulus was presented and participants’ behavior was observed.

#### 5.2.1. Perceived fear appeal design

The manipulation checks that address the subjectively perceived design of the fear appeals show that the manipulations were successful. Participants compared the threat level of the low and high threat messages on a bipolar 5-point Likert scale with the middle option representing equally severe threats (coded as 3). The high-threat message was rated as more severe than the low-threat message (95% CI: 4.50, 4.59). Saliency was compared between the low and high saliency messages on a bipolar 5-point Likert with the middle option representing messages that stand out equally (coded as 3). The saliency of the high saliency message was rated as standing out more than the low saliency message (95% CI: 4.75, 4.82). The readability of the text in the low saliency condition, which was rated on a unipolar 5-point Likert scale (1 = Not at all difficult, 5 = Very difficult), showed that readability was good, being that for the most part participants indicated that the text was not at all difficult to read (95% CI: 1.80, 1.90).

#### 5.2.2. Perceived fear appeal effectiveness

The PMT constructs employed in this study are reported in Appendix D. These items have been previously used in the ISec context (Boss et al. 2015). The relevant subset of the constructs used to measure perceived fear appeal effectiveness is shown in Table 2. The scale measuring *severity* showed an unacceptably lower internal consistency (Cronbach’s α = 0.59) than those measuring *vulnerability, fear,* and *intention* (α = 0.71, 0.93, 0.77). We, therefore, excluded the construct *severity* from further analysis.

First, we analyze the treatment effect regarding our control variables. The analysis suggests that the treatments successfully manipulated *fear* and *intention*.

**Figure 1.**
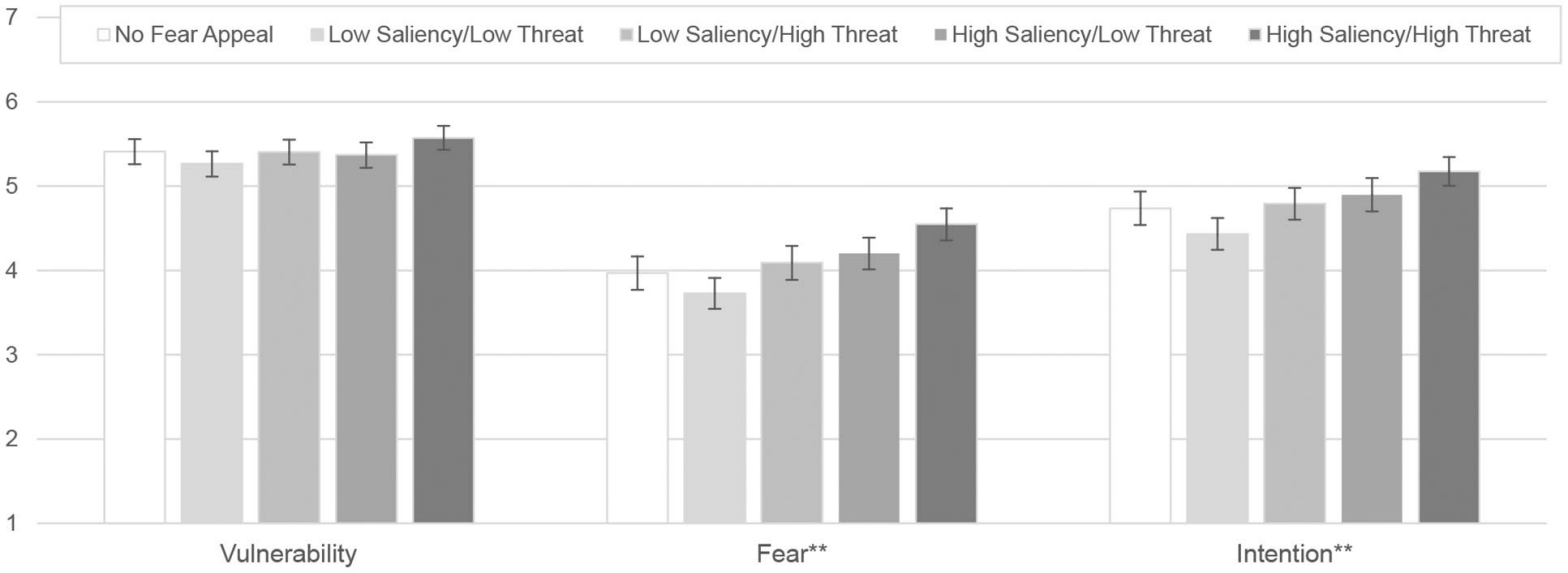
Treatment effects on selected PMT constructs (**p <.001; 95% CIs).

Figure 1 shows the mean values for the constructs used to measure fear appeal effectiveness. The high saliency/high threat treatment shows significantly higher levels of *fear* and *intention* than the low saliency/low threat treatment. Although not statistically significant, it is interesting to observe that the mean levels for low saliency/low threat are below those of the no fear appeal condition for all constructs. Mean differences among the groups were analyzed for each construct. Results of the ANOVA show that statistically significant differences exist for *fear* and *intention* (F(4,1481) = 10.02, p < 0.001, f = 0.16; F(4,1481) = 8.26, p < 0.001, f = 0.15). No significant differences exist for *vulnerability* (F(4,1481) = 2.29, p = 0.57, f = 0.07).

**Figure 2.**
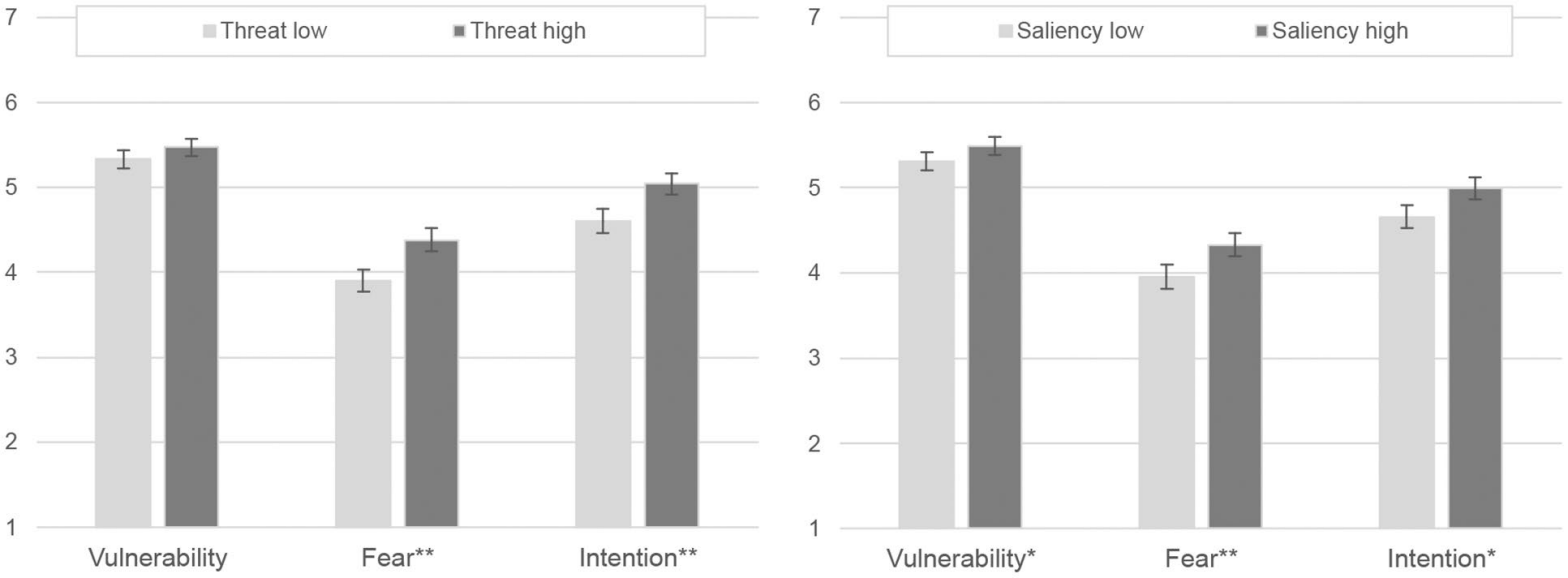
Effects of independent variables on selected PMT constructs (*p <.05, **p <.001; 95% CIs).

Second, we analyze the effects for each of our two main independent variables separately. The analysis suggests that the factor *threat* successfully manipulated *fear* and *intention*, and the factor *saliency* also successfully manipulated *vulnerability*. Figure 2 shows the PMT constructs’ mean values separately for the two independent variables *threat* and *saliency* (without participants in the control condition). ANOVA results show that a high threat level results in significantly higher values for *fear* and *intention* compared to a low threat level (F(1,1205) = 24.19, p < 0.001, f = 0.14; F(1,1205) = 21.28, p < 0.001. f = 0.13). No significant differences exist for *vulnerability* (F(1,1205) = 3.68, p = 0.55, f = 0.05). A high saliency level results in significantly higher levels of *vulnerability*, *fear* and *intention* compared to low saliency levels (F(1,1205) = 5.63, p < .05, f = 0.07; F(1,1205) = 14.46, p < 0.001, f = 0.11; F(1,1205) = 12.40, p < .05, f = 0.10).

### 5.3. Results of hypothesis tests

**Figure 3.**
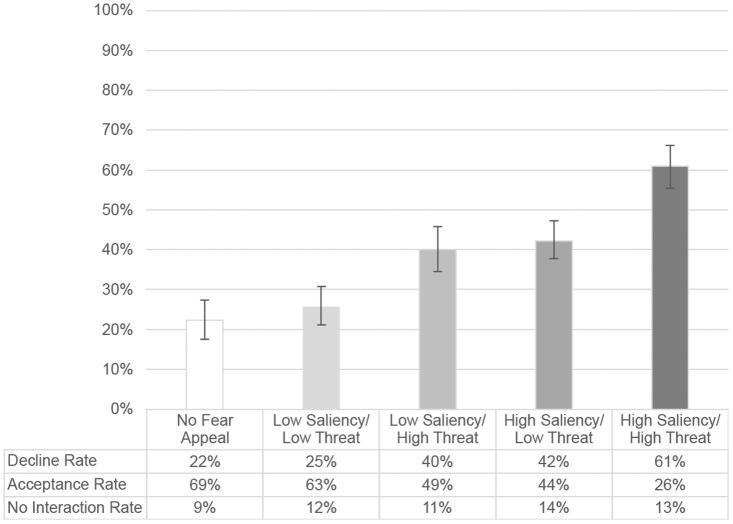
Decline rates across the experimental conditions (95% CIs).

The focal dependent variable for this study is the cookie *decline rate,* that is, the proportion of participants who chose to decline cookies. The *decline rates* per experimental condition are indicated in Figure 3. While only 22% of the participants declined the use of cookies in the control condition where a fear appeal was absent, 61% did so in the high saliency/high threat condition. No significant differences in decline rates were found between participants in the control condition and the low saliency/low threat condition on the one hand, and between the low saliency/high threat (40%) and the high saliency/low threat condition (42%) on the other.

**Figure 4.**
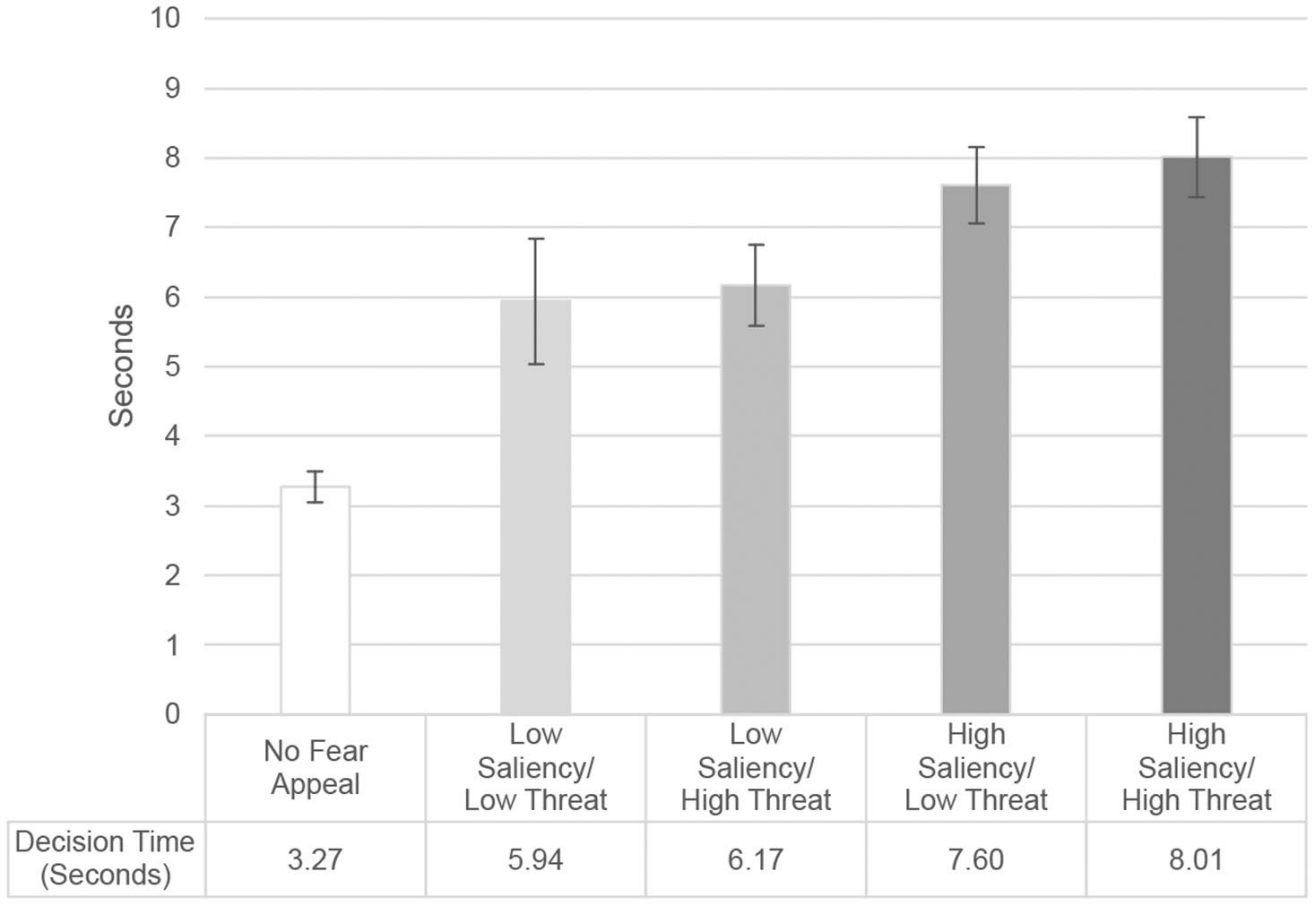
Decision time across the experimental conditions (95% CIs).

Figure 4 shows the time (in seconds) that it took participants who interacted with the cookie banner to decide whether to accept or decline the use of cookies (Participants who did not interact with the cookie banner and thus made no decision are excluded from this analysis). While it took participants in the control condition without a fear appeal only 3.3 seconds on average to make a decision, participants in the high saliency/high threat condition required 8 seconds on average to arrive at a choice. The overall pattern of results regarding reaction times as visualized in Figure 4 indicates that participants in the high saliency conditions paid significantly more attention to the fear appeal than participants in the low saliency conditions. Moreover, there is no significant difference in decision times between the low-threat and the high-threat conditions. These results suggest that saliency is the primary driver of attention in our context, which is in line with the previous literature.

A logistic regression analysis with the individual decisions to decline as the dependent variable with two levels (yes = 1, no = 0) was conducted to test our hypotheses. The results indicate that the model is highly significant (c^2^(4, n = 1486) = 124.25, p < .001).

Table 3 shows the corresponding regression results. The availability of a fear appeal per se does not appear to have a significant effect (p = 0.35). However, the individual coefficients *threat* and *saliency* are highly significant (p < .001), and the relative probability to decline increases by 96% (Exp(B) = 1.96) if the threat level is high rather than low and by 114% (Exp(B) = 2.14) if the saliency level is high rather than low. We did not observe a significant interaction between *threat* and *saliency* (p = 0.73), however. Other models were tested that additionally take the demographic variables and the PMT constructs into account. They show that the significant effects of threat and saliency are robust to the inclusion of further control variables and that the effect of saliency is consistent, though not statistically significant, larger than the effect of threat.

Therefore, the data support the first two confirmatory hypotheses:

- Participants exposed to fear appeals in the high-threat conditions are significantly more likely to decline the use of cookies as compared to participants exposed to fear appeals in the low-threat conditions (Hypothesis 1).
- Participants exposed to fear appeals in the high saliency conditions are significantly more likely to decline the use of cookies as compared to fear appeals in the low saliency conditions (Hypothesis 2).

The data do not confirm hypothesis 3: the saliency of the fear appeal content did not moderate the effectiveness of the described threat. Instead, the effects of saliency and threat appear to be additive.

## 6. Discussion

Previous studies have demonstrated that the visual appearance of warnings can increase warning perception (Sunshine et al. 2009) and reduce the effects of habituation (Vance et al. 2018). Also, researchers have shown that different threat levels in warnings can lead to different levels of individual protection behavior (Boss et al. 2015).

Our study extends previous research by demonstrating that individuals’ decisions to protect themselves *simultaneously* depend on both the objective threat and the saliency with which the threat is communicated. This is an important finding insofar as it makes clear that the effectiveness of a warning to motivate protection behavior can depend on visual design features at least as much as it depends on the threat level that it informs about. Also, with up to more than one-third of the users changing their behavior in response to changes in these two factors, the corresponding effect sizes are not trivial. Contrary to our expectations, we found no evidence for an interaction between saliency and threat. In absolute terms, high saliency yielded a 21% increased decline rate between low and high threat, compared to 17% in the low saliency conditions. However, this increase is not statistically significant. A potential explanation could be that our low saliency conditions were already relatively highly salient. One indicator in this respect is the decision time, which is already significantly increased in both low saliency conditions compared to the control condition (no fear appeal). Furthermore, as color (like size) is a feature guiding visual attention (Wolfe and Horowitz 2017), the presentation of the warning text in red (as opposed to using the same grey color as was used for the other text) in all treatment conditions might have strongly decreased the saliency difference between the low and high saliency conditions manipulated by the font size. Consequently, our saliency manipulation might have been confounded or too weak to allow for the detection of an interaction effect between saliency and threat.

### 6.1. Implications for research and practice

Previous studies that employed fear appeals, primarily studies from ISec, silently adopt the assumption of rational decision-making unbiased by arbitrarily selected visual design features. In other words, these studies assume that users form a protection motivation and show a corresponding behavioral reaction based solely on the informational content of a fear appeal. Other studies explore determinants of behavior related to the informational content, such as personal relevance, abstractness, or gain and loss framing (Johnston et al. 2019; Schuetz et al. 2020; Seo and Park 2019). At the same time, there is ample evidence from risk research, psychology, and other research areas, showing that human decision-making is often dependent on objectively irrelevant, subtle contextual cues not directly related to the content, which a rational decision agent would not take into account for making a choice. Similar findings were made for warnings about health or environmental hazards, such as for household chemicals, where consumers considered hazard-irrelevant product design features to inform their risk perception (Bearth, Miesler, and Siegrist 2017; Buchmüller et al. 2022). Such an example is the saliency with which information is presented. The current research bridges between these research areas by addressing both the effect of a fear appeal’s potentially relevant information content and its saliency as an objectively irrelevant design feature. This research also potentially helps to explain the varying and sometimes contradictory observations of the effectiveness of fear appeals reported in ISec (Schuetz et al. 2020). This research also stresses the importance to involve other sciences, aside from ISec, in research on digital risks and information security. The vast knowledge about human judgment and decision-making under uncertainty gained in other areas could be useful, as it is plausible that similar mechanisms would apply in digital environments.

Further, our study also contributes to the field of human-computer interaction. Previous research has already shown that salient visual stimuli attract more attention and are better recalled than less salient stimuli (e.g. Ebert, Ackermann, and Scheppler 2021; Veas et al. 2011), or that making one option more salient than other options can lead to an increase in the proportion of people choosing that particular option (Krajbich, Armel, and Rangel 2010; Krajbich and Rangel 2011; Milosavljevic et al. 2012; Rangel, Camerer, and Montague 2008; Shimojo et al. 2003). Our results expand on previous findings by showing that saliency may not only affect choice behavior when it is used as a design feature of the choice options themselves but also when it is used as a design feature of the information to which the choice options refer. This insight can also be transferred to other areas where warning information can be made more salient (e.g. use instruction on biocides as environmental risk mitigation measure; health warnings and safety information on household chemicals, car alerts). Saliency might be particularly relevant in areas where resources available for warnings (i.e. attention, time, motivation) are particularly low, due to prioritization or distractions.

Cookies can be used to track user behavior, and in 2009, the ‘EU Cookie Directive’ (2009/136/EC) changed Article 5(3) of the ePrivacy Directive (2002/58/EC) to state that ‘the storing of information … in the terminal equipment of a … user’ is only allowed if the individual ‘has given his or her consent, having been provided with … information … about the purposes of the processing’ (DIRECTIVE 2009/136/EC OF THE EUROPEAN PARLIAMENT AND OF THE COUNCIL 2009). This led to websites worldwide moving to prominently display consent notices (referred to as ‘cookie banners’), informing users about the use of cookies and asking for their consent. Despite its name, the EU Cookie Directive covers all forms of online tracking technology (such as device fingerprinting, for instance) and thus does not only apply to cookies in the narrow sense. However, while many websites are forced to display cookie banners, the way in which these cookie banners are visually designed is hardly regulated at all. At the same time, website owners clearly have an incentive to increase the number of visitors who accept cookies and therefore also use means of visual design (Cofone 2017).

As introduced in this article, new regulations have led to an increase in privacy information provided to users, among others via cookie banners (DIRECTIVE 2009/136/EC OF THE EUROPEAN PARLIAMENT AND OF THE COUNCIL 2009). Additionally, these new regulations expect consumers to judge and make decisions. Insights from this article and other research on decision-making under uncertainty could contribute to a better understanding of how these decisions are made and how users could be warned about their decisions’ possible negative outcomes. Using saliency as a design feature by altering visual attributes, such as size or color, to evoke heightened (or in some situations, perhaps lowered) perception of threats, may be effective in altering behavior.

### 6.2. Limitations and future research

In this study, we investigated the concurrent effects of a warning’s content in terms of threat severity and the warning’s saliency on privacy protection behavior in the context of cookie banners. To do this we varied saliency via varying the warning message’s font size and varied threat severity via referring to inconsequential information or an actual privacy-invasive data practice. We measured individuals’ decisions to decline the use of cookies on a website that participants were led to believe was real, and which was designed to look authentic, but was fictitious. As a natural consequence of these specific choices regarding experimental design and study context, there are several limitations that we would like to discuss, and which may stimulate future research.

As we cannot rule out that our saliency manipulation (font size) was confounded by design choices (text color), follow-up research may address the question of whether an interaction effect may be present when confounding effects are absent. Directly related is the question to which extent other ways of altering saliency, such as changing color or introducing movement, would be differentially effective as compared to manipulating font size.

We can think of several contextual factors that may diminish saliency effects or eliminate them completely under certain conditions. For instance, it has been shown that situational factors, such as cognitive load or the duration of a stimulus presentation, can mitigate the effect of saliency on behavior (Milosavljevic et al. 2012). Presumably, the saliency of information may also have a differential impact on behavior depending on how much prior knowledge a recipient of the information has of the issue in question. More generally, the way information is processed cognitively in a particular situation may play a considerable role as well. For instance, research-based on dual-process theories such as the Elaboration Likelihood Model (Petty and Cacioppo 1986) has shown that persuasive messages may be processed very differently depending on the recipients’ motivation and cognitive capacity. For example, participants of an IT security awareness training may be highly motivated and have enough cognitive capacity to recognize a simulated phishing warning in their e-mail client. In such a setting with highly vigilant decision-makers, saliency may matter considerably less than in a setting where consumers make trivial everyday decisions mostly guided by habit.

Furthermore, we can only speculate if the pattern of results we found would also hold in other choice contexts using other kinds of stimulus materials. We decided for an adult website as the choice context and session replay with third parties as the privacy practice at hand to increase the likelihood that the risk of privacy intrusion in the high-threat condition was indeed perceived as highly severe. It is an open question whether the same study design choices would lead to the same results given a different choice context, such as a mainstream online shop, for instance. A user’s evaluation of privacy risks is to some extent context-specific (Ebert, Ackermann, and Heinrich 2020), such that the perception of the severity of a particular privacy practice may vary depending on the choice context. However, we would argue that the pattern of results we found in our study would also hold in other contexts, given that the threat that is conveyed in a warning message is perceived as severe as was the case for the subjects in our study.

We also investigated the effect of saliency on protection behavior in a cross-sectional study, so we cannot rule out that the effect would diminish over time if users were confronted with the warning repeatedly across multiple situations.

Finally, our study was designed to investigate the relative size of effects that the informational content and visual saliency of a warning message have on protection behavior irrespective of the structural nature of the effect paths. Hence, it remains an open question whether these two factors act simultaneously or sequentially – and, in case of the latter, in which sequence the effects operate. For instance, does the visual saliency of a message increase the attentiveness towards the informational content such that it is elaborated more deeply, or do individuals first process the informational content which is then cognitively assigned more significance due to the visual saliency of its appearance? Future research may address questions of this kind to investigate the nature of the cognitive processes that lead to the behavioral results we observed in our study.

## 7. Conclusion

To conclude, we hope that this study provides helpful impetus to the continuing research on the design of effective security warnings. Our results have important implications for designers of digital warnings that need to communicate with users efficiently and effectively as well as regulators that want to enforce effective warnings. Our findings can be applied in various application areas such as information security, digital health (e.g. health warnings on smartwatches), or cars (e.g. low battery warning on navigation panel). We demonstrate that a warning message’s informational content and its saliency are equally important and that consequently both these factors should be paid attention to when designing user dialogs.

## Figures and Tables

**Figure A1.**
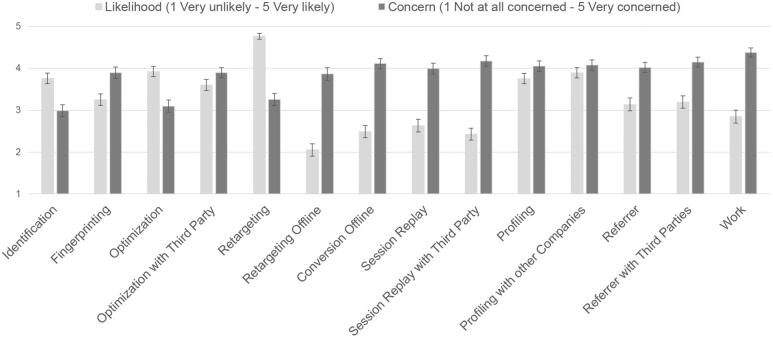
Cookie tracking likelihood and concern rated by the participants (with 95% CIs).

**Figure A2.**
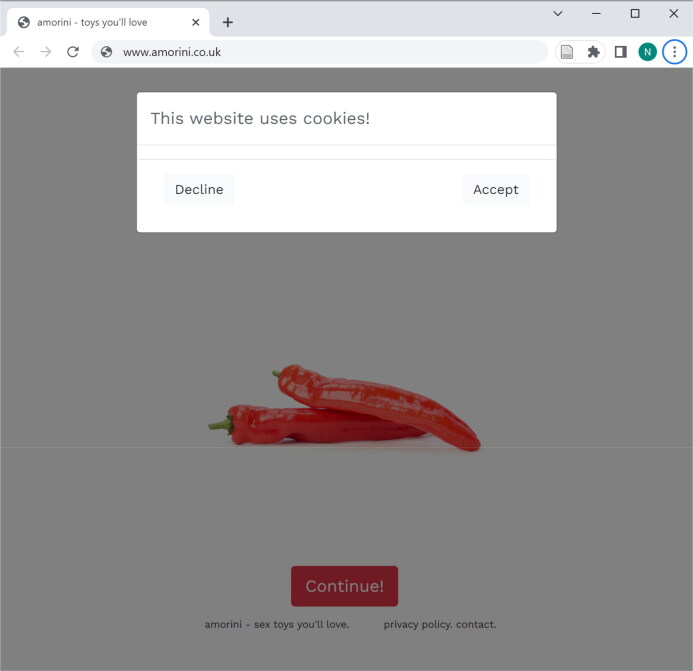
Stimulus homepage as displayed to the participants.

## Data Availability

The raw data are available via https://osf.io/vr6b7/

## Appendix A. Cookie tracking survey

In October 2020 we asked participants to complete a “Cookie Tracking” survey via prolific, a subject pool for academic research. Participants had to reside in the UK and were provided with a small monetary compensation for completing the survey. The final sample included 290 participants. The age of the participants ranged from 18 to 71 (M = 33.6). Of all respondents 71.7% were female. Sixty per cent had at least completed a bachelor’s degree or comparable professional degree. More than 70% of the participants reported to have seen cookie banners on 75% or more of websites they had visited in the past week before the survey.

We confronted participants with different cookie tracking practices to find out which of these had the potential to raise privacy concerns. We asked participants to evaluate the likelihood of certain cookie tracking practices on a website as well as the resulting level of concern on a 5-point Likert scale. We used a shopping website as the specific context for cookie tracking because this segment uses a broad range of tracking techniques.

Figure A1 shows the rating of the cookie tracking practices by the participants. A simple identification of the user was considered more likely than, for example, session replay with third parties. However, the latter raised more concern than identification. For the main experiment, we were interested in a practice that was (a) considered realistic in our limited experimental setting, (b) raised a high level of concern, and (c) was considered less likely. Due to criteria (a) we rejected retargeting offline, conversion offline and work-related tracking. Subsequently, due to (b) and (c), we decided use session replay with third parties as a high threat in the main experiment.

**Table A1.** Demographics of the sample in the main experiment.

Condition	No Fear Appeal	Low Saliency/ Low Threat	Low Saliency/ High Threat	High Saliency/ Low Threat	High Saliency/ High Threat
Participants	279	314	292	284	317
Age (Years)					
Mean	37.46	36.36	36.98	35.69	36.53
Sd	13.88	13.22	13.34	12.72	13.43
Gender (%)					
Male	41	45	37	42	44
Female	59	54	62	58	55
Other	0	1	1	0	0
Highest level of education (1 = None, 2 = Secondary education, 3 = Pre-University/Further Education, 4 = Bachelor or comparable, 5 = Master or comparable, 6 = PhD or comparable)					
Median	4	4	4	4	4

## Appendix D. Measures for PMT constructs (Table A2)

**Table A2.** Measures for PMT constructs.

Construct	Item
Perceived severity (Milne, Orbell, and Sheeran 2002)	If I were to be tracked by the website, I would suffer a lot of pain. (strongly disagree - strongly agree)
	Being tracked by the website is unlikely to cause me major problems.^a^ (strongly agree - strongly disagree)
Vulnerability (Milne, Orbell, and Sheeran 2002)	I am unlikely to be tracked by the website when I accept cookies.^a^(strongly agree - strongly disagree)
	My chances of being tracked by the website when I accept cookies are (not at all high - very high)
Fear (Milne, Orbell, and Sheeran 2002)	I would be worried about being tracked by the website if I accept cookies. (strongly disagree - strongly agree)
	I would be frightened about being tracked by the website if I accept cookies. (strongly disagree - strongly agree)
	I would be anxious about being tracked by the website if I accept cookies. (strongly disagree - strongly agree)
	I would be scared about being tracked by the website if I accept cookies. (strongly disagree - strongly agree)
Response Efficacy (Milne, Orbell, and Sheeran 2002)	Declining cookies is a good way to reduce the risk of being tracked by the website. (strongly disagree - strongly agree)
	If I were to decline cookies, I would lessen my chances of being tracked by the website. (strongly disagree - strongly agree)
Self-efficacy (Johnston and Warkentin 2010)	Declining cookies is easy. (strongly disagree - strongly agree)
	Declining cookies is convenient. (strongly disagree - strongly agree)
	I am able to decline cookies without much effort. (strongly disagree - strongly agree)
Response cost (Woon, Tan, and Low 2005)	Declining cookies decreases the convenience afforded by the website. (strongly disagree - strongly agree)
	There is too much work associated with trying to increase privacy by declining cookies on the website. (strongly disagree - strongly agree)
	Declining cookies requires considerable investment of effort other than time. (strongly disagree - strongly agree)
	Declining cookies is time consuming. (strongly disagree - strongly agree)
Maladaptive rewards (Myyry et al. 2009)	Accepting cookies saves me time. (strongly disagree - strongly agree)
	Accepting cookies brings me advantages. (strongly disagree - strongly agree)
	Accepting cookies keeps me from being confused. (strongly disagree - strongly agree)
	Declining cookies limits the functionality of the website. (strongly disagree - strongly agree)
Intention (Milne, Orbell, and Sheeran 2002)	I intend to decline cookies from the website in the future. (strongly disagree - strongly agree)
	I do not wish to decline cookies from the website in the future.^a^ (strongly agree - strongly disagree)

^a^ reverse-coded items.

**Table 1.** Illustration of experimental stimuli in each condition.

No fear appeal (Control)		
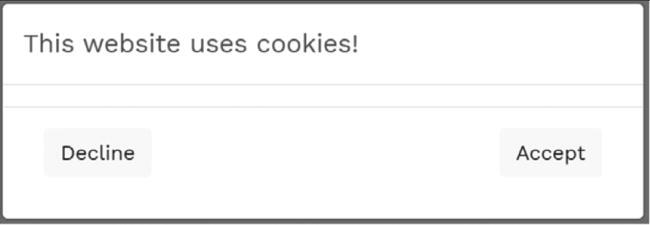
	Low Saliency	High Saliency
Low Threat		
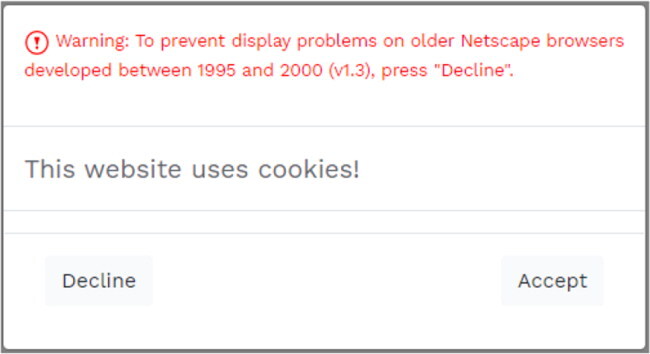
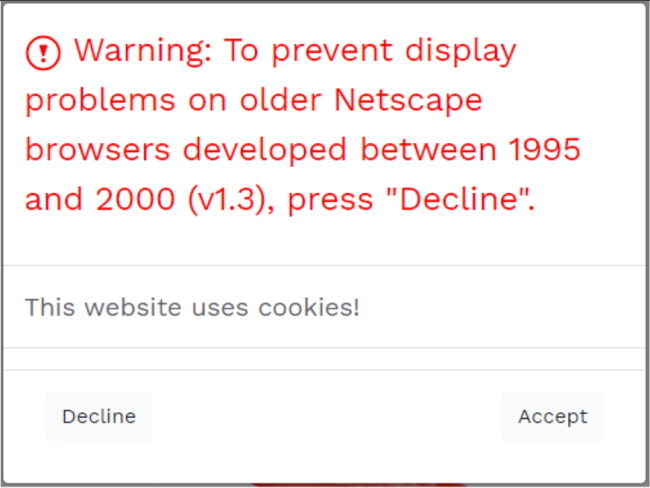
High Threat		
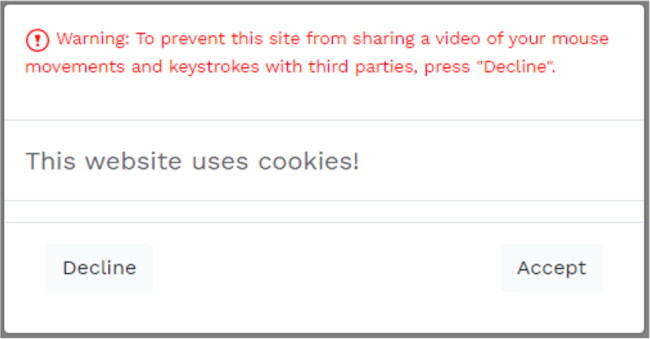
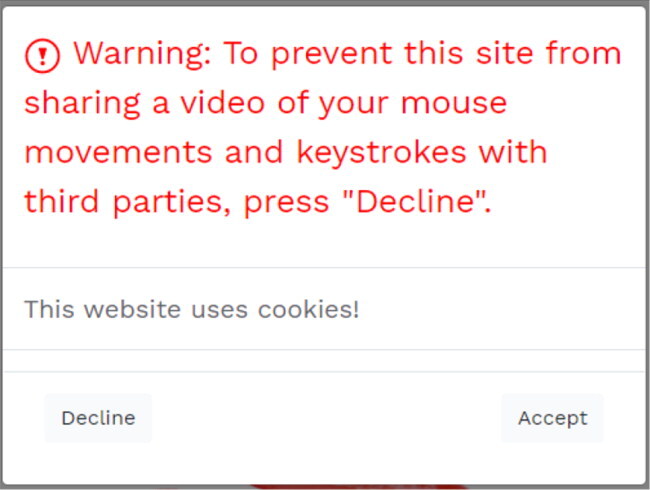

**Table 2.** Manipulation checks measured immediately after the experimental task.

Experimental treatment	Manipulation check	Mean (SD)	Statistical test
Perceived fear appeal design			
Threat level	Five-point bipolar Likert scale from “(A) more severe than (B)” = 1 to “(B) more severe than (A)” = 5: Which of the two warnings in a cookie banner on a website do you feel is referring to a more severe threat to your privacy? Warning: To prevent display problems on older Netscape browsers developed between 1995 and 2000 (v1.3), press "Decline". Warning: To prevent this site from sharing a video of your mouse movements and keystrokes with third parties, press "Decline".	4.55 (0.89)	Confidence interval
Saliency level	Five-point bipolar Likert scale from “(A) stands out more than (B)” = 1 to “(B) stands out more than (A)” = 5: Which of the two warnings in a cookie banner on a website stands out more?	4.79 (0.66)	Confidence interval
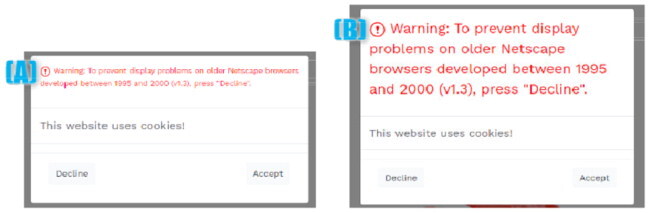
Readability (Low Saliency)	Five-point Likert scale from “Not difficult at all” = 1 to “Very difficult” = 5: How difficult is the red warning text for you to read?	1.85 (1.04)	Confidence interval
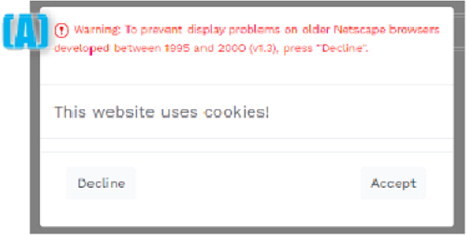
Perceived fear appeal effectiveness (measured before fear appeal design checks)			
Perceived severity (Milne, Orbell, and Sheeran 2002)	Seven-point Likert from “Strongly disagree” = 1 to “Strongly agree” = 7: If I were to be tracked by the website, I would suffer a lot of pain. Being tracked by the website is unlikely to cause me major problems.^a^	2.50 (1.51) 3.65 (1.63)	ANOVA
Perceived vulnerability (Milne, Orbell, and Sheeran 2002)	I am unlikely to be tracked by the website when I accept cookies^a^ (seven-point Likert from “Strongly disagree” = 1 to “Strongly agree” = 7): My chances of being tracked by the website when I accept cookies are… (seven-point Likert from “Not high at all = 1 to “Very high” = 7)	5.41 (1.47) 5.40 (1.47)	ANOVA
Fear (Milne, Orbell, and Sheeran 2002)	Seven-point Likert from “Strongly disagree” = 1 to “Strongly agree” = 7: I would be worried about being tracked by the website if I accept cookies. I would be frightened about being tracked by the website if I accept cookies. I would be anxious about being tracked by the website if I accept cookies. I would be scared about being tracked by the website if I accept cookies.	4.39 (1.84) 3.85 (1.87) 4.23 (1.89) 3.97 (1.88)	ANOVA
Intention (Milne, Orbell, and Sheeran 2002)	Seven-point Likert from “Strongly disagree” = 1 to “Strongly agree” = 7: I intend to decline cookies on the website in the future. I do not wish to decline cookies on the website in the future.^a^	4.66 (1.93) 4.95 (1.76)	ANOVA

^a^ reverse-coded items.

**Table 3.** Logistic regression for decline rate.

	B	S.E.	Wald	df	Sig.	Exp(B)
Constant	−1.25	0.14	75.68	1	0.000	0.29
Fear Appeal	0.18	0.19	0.86	1	0.354	1.20
Threat	0.67	0.18	14.49	1	**0.000**	1.96
Saliency	0.76	0.18	18.56	1	**0.000**	2.14
Threat x Saliency	0.08	0.24	0.12	1	0.729	1.09
